# A systematic review of the content and delivery of clinical knowledge in orthodontic postgraduate programs

**DOI:** 10.1186/s12909-025-07361-x

**Published:** 2025-05-20

**Authors:** Martin Baxmann, Krisztina Kárpáti, Zoltán Baráth

**Affiliations:** 1 Department of Orthodontics, Faculty of Education and Research, DTMD University, 9516 Wiltz, Luxembourg; 2 Department of Orthodontics and Pediatric Dentistry, Faculty of Dentistry, University of Szeged, Szeged, 6720 Hungary; 3 Department of Prosthodontics, Faculty of Dentistry, University of Szeged, Szeged, 6720 Hungary

**Keywords:** Orthodontic postgraduate education, Clinical knowledge, Teaching methods, Educational content, Clinical competence

## Abstract

Orthodontic postgraduate education plays a critical role in developing clinical competence, yet substantial variability exists in how programs structure, deliver, and assess clinical knowledge. This systematic review aimed to evaluate the content and delivery of clinical knowledge in orthodontic postgraduate programs and their reported impact on trainee competence. The review followed PRISMA 2020 guidelines and a pre-specified protocol. A comprehensive search was conducted across PubMed, Cochrane Library, ERIC, and CINAHL for studies published between January 2000 and December 2024. Gray literature and reference lists were also screened. Eligible studies included randomized controlled trials, cohort studies, and cross-sectional studies involving postgraduate orthodontic students or educators, with interventions related to instructional strategies or clinical knowledge delivery. Qualitative studies and those focused solely on undergraduate or continuing education were excluded. Following duplicate removal, 104 records were screened, and 30 studies met the inclusion criteria after full-text assessment. Study selection and data extraction were performed independently by two reviewers, with discrepancies resolved by a third. Risk of bias was assessed using the Newcastle–Ottawa Scale, and quality of evidence was evaluated using the GRADE framework. Due to heterogeneity in study design and outcome measures, a narrative synthesis was conducted. Instructional strategies included didactic lectures, problem-based learning, scenario-based simulations, digital learning platforms, and structured assessments such as Mini-CEX and OSCE. Feedback-oriented and interactive methods were consistently associated with improvements in clinical reasoning, procedural skills, and learner confidence. However, substantial variability existed in assessment formats and feedback implementation across institutions. Newcastle–Ottawa scores indicated generally strong selection and outcome measurement, though comparability was often limited. GRADE assessments showed most studies were of moderate quality due to inconsistency or imprecision. These findings highlight the need for harmonized, evidence-based frameworks to guide the delivery and assessment of clinical knowledge in orthodontic postgraduate education, while allowing for flexibility based on institutional context. Efforts to standardize assessment strategies and integrate technology-enhanced learning may support improved competence development across diverse programs.

## Introduction

Postgraduate orthodontic education is a complex and varied field, shaped by regional standards, institutional philosophies, and the evolving landscape of dental technologies [1]. These programs typically consist of a combination of theoretical instruction, clinical practice, research, and continuous assessment aimed at developing both foundational knowledge and advanced clinical skills [2]. However, the standards and methods employed in orthodontic training are not universally consistent; they often reflect the regulatory frameworks and professional expectations of different countries [3]. For example, some regions require a higher number of clinical hours and diverse patient cases, while others might prioritize research and academic achievement. This variability may lead to differences in clinical competence and readiness among graduates, highlighting the need for harmonized educational standards that ensure all orthodontists are adequately prepared, regardless of where they train. Therefore, this systematic literature review aimed to answer the question: How is clinical knowledge in orthodontic postgraduate programs delivered, and how does this impact the clinical competence and patient outcomes of orthodontic practitioners? Understanding how clinical knowledge is delivered as well as its results are essential for evaluating the impact of educational practices on clinical competence and patient outcomes, as it underscores the broader implications of how clinical knowledge is structured and delivered in orthodontic postgraduate programs.

## Methods

This systematic review followed a pre-specified methodology to ensure rigor in study selection, data extraction, and analysis. A protocol was submitted to PROSPERO but was not accepted due to the absence of a defined health intervention. No deviations from the original study design occurred. The eligibility criteria for this review were guided by the PICOT framework to ensure a structured and systematic approach to study selection. The population of interest comprised postgraduate orthodontic students and educators, with interventions encompassing didactic lectures, simulation-based training, digital learning platforms, mentorship programs, competency-based assessments, and hybrid learning models. Comparisons were made between traditional instructional methods and alternative or emerging educational approaches. The primary outcomes assessed included measures of clinical competence, knowledge retention, and skill development. To ensure the findings reflected current pedagogical practices, the review was restricted to studies published between January 1, 2000, and December 31, 2024.

Research focusing exclusively on undergraduate orthodontic programs, other dental specialties, or continuing education courses for practicing clinicians was excluded. Articles not available in English were also excluded to maintain consistency in data extraction and interpretation. The inclusion of randomized controlled trials (RCTs), cohort studies, and cross-sectional studies allowed for the evaluation of educational interventions using measurable clinical outcomes. Studies incorporating qualitative methodologies were excluded because they did not provide standardized, comparable measures of clinical competence. Although this review primarily examined educational interventions rather than direct patient outcomes, the selected studies evaluated their impact on clinical competence, skill acquisition, and decision-making. These competencies are critical components of patient care, as postgraduate orthodontic education is structured to ensure that residents develop the necessary skills to perform evidence-based treatments safely and effectively.

A comprehensive literature search was conducted across PubMed, Cochrane Library, ERIC, and CINAHL. The search strategy was developed using Medical Subject Headings (MeSH) terms and Boolean operators to refine results and ensure comprehensive retrieval of relevant studies. Search terms included"orthodontic postgraduate education"AND"clinical competence","teaching strategies"AND"skill development", and"educational interventions"AND"orthodontic training", applied systematically across all databases. Reference lists of included studies were manually reviewed to identify additional relevant literature. To broaden the scope of the review, gray literature sources such as conference proceedings, institutional reports, and government publications were screened for relevant studies not indexed in peer-reviewed databases. The final search was executed on September 24, 2024.

Study selection was performed independently by two reviewers (MB and KK), who screened titles and abstracts against predefined eligibility criteria. Full-text assessments were conducted independently by two reviewers, and discrepancies were resolved through discussion or, when necessary, consultation with a third reviewer (ZB). Data were extracted using a standardized form to ensure consistency, capturing study characteristics such as research design, descriptions of educational interventions, assessment methods, and primary clinical and educational outcomes. Extracted data were cross-checked between reviewers before final synthesis to ensure accuracy and reliability.

The risk of bias was assessed using the Newcastle–Ottawa Scale (NOS) for observational studies, evaluating factors such as participant selection, comparability of study groups, and outcome assessment methods [4]. To evaluate the overall quality of evidence, the Grading of Recommendations, Assessment, Development, and Evaluation (GRADE) framework was applied, assessing methodological limitations, consistency of findings, precision of outcome measures, and confidence in effect estimates [5]. These assessments were incorporated into the interpretation of findings to ensure that results account for study limitations.

A narrative synthesis was conducted to identify key themes and methodological trends across the included studies. Given the heterogeneity in study designs, sample sizes, and outcome measures, a meta-analysis was not feasible. Results were analyzed based on teaching methodologies, geographic variation, and assessment strategies, with an emphasis on recognizing the influence of institutional and regional differences on postgraduate training approaches.

## Results

A total of 374 records were identified through database searches. After removing 270 duplicate records, 104 unique records remained for screening. Of these, 39 records were excluded after title and abstract screening due to irrelevance. Sixty-five reports were sought for retrieval. Upon full-text assessment, 35 reports were excluded: 32 due to irrelevance to the study's focus, one due to the lack of full text, and two due to unsuitable methods. A full list of excluded full-text records was not retained; however, reasons for exclusion are summarized by category in accordance with PRISM 2020 guidelines. Ultimately, 30 studies met the inclusion criteria and were included in the review (Table 1).

**Table 1:** Studies included in the review

Author(s)	Year of Study	Sample Size	Population Description	Intervention or Exposure	Outcomes Measures	Results
Al-Jewair & Kumar	2019	24	First-year orthodontic residents at a US dental school	Mini-Clinical Evaluation Exercise as a work-based assessment	Clinical performance across seven domains including interviewing skills and clinical judgment	Mean scores improved over four encounters; satisfaction rates were high among residents and evaluators
Allanqawi et al	2023	168	Graduates of postgraduate orthodontic programs	Various aspects of postgraduate orthodontic education	Different educational approaches and experiences in orthodontic training	Details of various orthodontic postgraduate programs across different countries
Allareddy et al	2019	39	Orthodontic residency programs in the US	Survey assessing features of an excellent orthodontic residency program, focusing on faculty, education, and resident/alumni aspects	Key features like faculty numbers, board certification rates, resident exposure to diverse treatments, clinical session coverage, and resident/alumni outcomes	Identified attributes for an excellent program, such as adequate full-time faculty, high board certification rates, sufficient clinical exposure, and strong resident performance in board examinations
Aly et al	2003	100	Undergraduate and postgraduate dental students at Katholieke Universiteit Leuven	Interactive multimedia courseware for orthodontic self-study	Student enthusiasm and effectiveness of multimedia learning	High enthusiasm among students; multimedia seen as effective for self-study
Aly et al	2012	24	Postgraduate orthodontic students	Effect of multimedia information sequencing on educational outcomes	Impact on learning efficiency and understanding of content	Students reported increased understanding and engagement with content
Arunachalam et al	2021	165	Orthodontic postgraduate students	Perception of digital learning tools in postgraduate orthodontics	Postgraduate students'feedback on the use of digital tools	Positive feedback from students on digital learning aids
Bearn & Chadwick	2010	18	Postgraduate dental students involved in problem-based learning in orthodontic training	Implementation of problem-based learning methods in orthodontic postgraduate education	Student engagement, knowledge retention, and satisfaction with the learning approach	Students reported enhanced engagement and understanding with problem-based learning
Bednar et al	2007	45	Orthodontic postgraduate students participating in distance learning interactive seminars	Application of distance learning seminars in orthodontic postgraduate programs	Effectiveness of distance learning seminars and student perceptions of interactive learning	Distance learning was effective but highlighted the need for improved interaction opportunities
Burk & Orellana	2013	75	Graduate orthodontic programs across North America, focusing on assessment and improvement	Survey of graduate orthodontic programs assessing program strengths and improvement areas	Program strengths, areas of improvement, and student satisfaction with program components	Identified key strengths in program structure and areas needing further development
Cure	2016	60	Orthodontic outreach program involving interprofessional education with postgraduate students	Interprofessional education within an orthodontic outreach program, evaluating student outcomes	Impact of interprofessional education on collaborative learning and clinical skills development	Positive impact on collaboration and clinical skills; enhanced student understanding reported
Fields et al	2007	46	Orthodontic residents in an advanced education program	Implementation of Objective Structured Clinical Examination (OSCE)	Proficiency in clinical skills across different years of training	Improvement in skills from first to second year; some skills did not reach proficiency
Hughes et al	2009	24	Orthodontic residents from multiple US programs focusing on learning styles	Assessment of preferred learning styles among orthodontic residents	Identification of predominant learning styles and implications for teaching methods	Identified visual and kinesthetic learning styles as predominant; recommended tailored teaching
Ireland et al	2013	9	Postgraduate students in orthodontics at the University of Bristol	Wiki topic teaching as a learner-centered approach	Student feedback on Wiki as a teaching tool	Positive feedback on team collaboration and self-directed learning through Wikis
Jamenis et al	2020	9	Orthodontic postgraduate students using Mini-CEX as a formative assessment	Mini-Clinical Evaluation Exercise (Mini-CEX) as an assessment tool	Clinical performance, communication skills, and student feedback	Significant improvement in performance with structured feedback
Johnson King et al	2022	42	Postgraduate students'perceptions on face-to-face vs online learning in orthodontics	Survey comparing student satisfaction with face-to-face and online learning modalities	Student satisfaction and perceived effectiveness of learning modes	Students showed a preference for face-to-face interaction but found online learning convenient
Kashif	2020	150	Postgraduate orthodontic students from various universities in the Middle East	Survey on educational preferences and challenges in postgraduate orthodontic training	Educational preferences, perceived gaps, and suggested improvements	Highlighted the need for improved resources and more interactive teaching approaches
Lishoy et al	2020	110	Second-year postgraduate students pursuing MDS in orthodontics during the COVID-19 lockdown	COVID-19 lockdown and its impact on orthodontic postgraduate students	Concerns about academic work, thesis, patient workload, and future practice	Majority of students felt their training was compromised due to the lockdown
Madhan et al	2011	51	Postgraduate orthodontic students, focusing on attitudes toward their education	Attitudes and concerns of postgraduate students in orthodontics	Student feedback on educational challenges and training quality	Many students expressed concerns about their training quality and career preparation
Masella & Meister	2002	55	Postdoctoral orthodontic educators and students discussing challenges in advanced education	Evaluation of challenges faced in postdoctoral orthodontic education	Challenges identified in curriculum design, student engagement, and technological adaptation	Highlighted need for updated curricula, improved faculty training, and better use of technology
Mcdonald et al	2000	37	Postgraduate (specialist) orthodontic programs in the UK and the USA	Survey of postgraduate orthodontic training programs	Program content, structure, and areas needing improvement	Differences in program structures and training quality across institutions
Mohan & Babu	2020	127	Postgraduate orthodontic programs in Chennai, focusing on digital study models	Survey on the use of digital study models and their trends	Knowledge and usage trends of digital models in postgraduate programs	Students found digital models to be beneficial, with trends suggesting increased usage
Nakornnoi et al	2023	32	Orthodontic residents in a postgraduate program in Thailand	Digital technology integration in orthodontic postgraduate programs	Perceptions of the usefulness of digital technology in training	Positive perceptions of digital technology, though some challenges were noted
Naser-ud-Din	2015	60	Orthodontic residents in a postgraduate scenario-based learning program	Implementation of scenario-based learning in orthodontic postgraduate training	Resident engagement, learning outcomes, and feedback on scenario-based methods	Scenario-based learning significantly improved critical thinking and practical skills
Oliver et al	2020	72	Orthodontic trainees discussing what they wish they had learned during training	Survey of orthodontic trainees'reflections on training gaps	Training gaps and areas needing more focus	Identified key areas trainees felt underprepared, including clinical decision-making and research skills
Papageorgiou et al. (2020)	2020	96	Postgraduate dental students from three universities assessed on evidence-based research knowledge	Survey on knowledge of evidence-based research and clinical trials	Correct responses to evidence-based research methodology questions	Overall correct answer rate was 456%, indicating moderate knowledge among students
Papageorgiou et al. (2022)	2022	46	Orthodontic postgraduate residents from four universities in Europe and the US on evidence-based methodology	Assessment of knowledge in evidence-based research methodology	Knowledge scores on clinical research design and critical appraisal	Overall knowledge score of 488%, highlighting areas needing further emphasis in curriculum
Polychronopoulou et al	2011	75	Orthodontic postgraduate students in Europe, focusing on biostatistics knowledge	Evaluation of biostatistics knowledge among orthodontic postgraduate students	Knowledge scores on statistical and methodological concepts	Moderate biostatistical knowledge; suggested need for improved statistical education
Shah & Cunningham	2009	38	Orthodontic postgraduate students using virtual learning environments in training	Use of virtual learning environments to enhance orthodontic education	Effectiveness of virtual learning environments on student engagement and knowledge retention	Positive impact on student learning and satisfaction, with calls for more interactive tools
Shinh et al	2017	799	Postgraduate orthodontic students in India, discussing academic challenges	Survey on challenges faced during orthodontic postgraduate training	Challenges in academic, psychological, and communication aspects	High levels of stress reported; recommendations for a more supportive learning environment
Stockstill et al	2011	54	Orthodontic residency programs focusing on teaching methods and effectiveness	Survey of orthodontic residency programs and teaching strategies	Evaluation of teaching methods and student satisfaction	Students expressed need for more structured and interactive teaching approaches

Most studies demonstrated strong selection criteria and reliable outcome measurements in the NOS. High scores in the selection category, seen in studies such as Cure [6] and Aly et al. [7], indicated well-defined and representative sample groups. Comparability scores were generally lower, with many studies scoring 1 out of 2, highlighting some limitations in controlling for confounding factors. Outcome scores consistently reflected well-defined and reliable outcome measures across studies, contributing positively to the overall quality (Table 2).

**Table 2:** Newcastle–Ottawa scale results

Author(s)	Selection	Comparability	Outcome	Total Score
Al-Jewair & Kumar (2019) [8]	3	1	2	6
Allanqawi et al. (2023) [1]	4	1	2	7
Allareddy et al. (2019) [9]	3	1	2	6
Aly et al. (2003) [10]	3	1	2	6
Aly et al. (2012) [7]	4	2	2	8
Arunachalam et al. (2021) [11]	3	1	2	6
Bearn & Chadwick (2010) [12]	3	1	2	6
Bednar et al. (2007) [13]	3	1	2	6
Burk & Orellana (2013) [14]	3	1	2	6
Cure (2016) [6]	4	2	2	8
Fields et al. (2007) [15]	3	1	2	6
Hughes et al. (2009) [16]	3	1	2	6
Ireland et al. (2013) [17]	4	2	2	8
Jamenis et al. (2020) [18]	4	1	2	7
Johnson King et al. (2022) [19]	3	1	2	6
Kashif (2020) [20]	3	1	2	6
Lishoy et al. (2020) [21]	3	1	2	6
Madhan et al. (2011) [22]	4	1	2	7
Masella & Meister (2002) [23]	3	1	2	6
Mcdonald et al. (2000) [24]	3	1	2	6
Mohan & Babu (2020) [25]	3	1	2	6
Nakornnoi et al. (2023) [26]	3	1	2	6
Naser-ud-Din (2015) [27]	4	2	2	8
Oliver et al. (2020) [28]	3	1	2	6
Papageorgiou et al. (2020) [29]	3	1	2	6
Papageorgiou et al. (2022) [30]	4	1	2	7
Polychronopoulou et al. (2011) [31]	3	1	2	6
Shah & Cunningham (2009) [32]	4	1	2	7
Shinh et al. (2017) [33]	3	1	2	6
Stockstill et al. (2011) [34]	3	1	2	6

The GRADE approach was employed to evaluate the overall quality of evidence from the included studies, assessing factors such as risk of bias, inconsistency, indirectness, imprecision, and publication bias. Of the 30 included studies, 6 were rated as high quality, 24 as moderate quality, and none as low or very low. Most studies received moderate ratings due to concerns related to inconsistency or imprecision in outcome reporting. These evaluations are summarized in Table 3.

**Table 3:** GRADE results

Author(s)	Risk of Bias	Inconsistency	Indirectness	Imprecision	Publication Bias	Overall Quality
Al-Jewair & Kumar (2019) [8]	Low	Low	Low	Low	Low	Moderate
Allanqawi et al. (2023) [1]	Moderate	Moderate	Low	Low	Moderate	Moderate
Allareddy et al. (2019) [9]	Low	Low	Low	Low	Low	Moderate
Aly et al. (2003) [10]	Moderate	Low	Low	Moderate	Low	Moderate
Aly et al. (2012) [7]	Low	Low	Low	Low	Low	High
Arunachalam et al. (2021) [11]	Low	Moderate	Low	Low	Moderate	Moderate
Bearn & Chadwick (2010) [12]	Moderate	Low	Low	Moderate	Low	Moderate
Bednar et al. (2007) [13]	Low	Low	Low	Low	Low	Moderate
Burk & Orellana (2013) [14]	Moderate	Low	Low	Moderate	Low	Moderate
Cure (2016) [6]	Low	Low	Low	Low	Low	High
Fields et al. (2007) [15]	Low	Low	Low	Low	Low	Moderate
Hughes et al. (2009) [16]	Low	Moderate	Low	Moderate	Low	Moderate
Ireland et al. (2013) [17]	Low	Low	Low	Low	Low	High
Jamenis et al. (2020) [18]	Low	Low	Low	Low	Low	High
Johnson King et al. (2022) [19]	Moderate	Moderate	Low	Moderate	Low	Moderate
Kashif (2020) [20]	Low	Moderate	Low	Moderate	Low	Moderate
Lishoy et al. (2020) [21]	Low	Low	Low	Moderate	Low	Moderate
Madhan et al. (2011) [22]	Low	Moderate	Low	Low	Moderate	Moderate
Masella & Meister (2002) [23]	Moderate	Low	Low	Moderate	Low	Moderate
Mcdonald et al. (2000) [24]	Low	Moderate	Low	Low	Moderate	Moderate
Mohan & Babu (2020) [25]	Low	Low	Low	Low	Low	Moderate
Nakornnoi et al. (2023) [26]	Low	Moderate	Low	Moderate	Low	Moderate
Naser-ud-Din (2015) [27]	Low	Low	Low	Low	Low	High
Oliver et al. (2020) [28]	Low	Low	Low	Moderate	Low	Moderate
Papageorgiou et al. (2020) [29]	Low	Low	Low	Moderate	Low	Moderate
Papageorgiou et al. (2022) [30]	Low	Low	Low	Moderate	Low	Moderate
Polychronopoulou et al. (2011) [31]	Low	Low	Low	Moderate	Low	Moderate
Shah & Cunningham (2009) [32]	Low	Low	Low	Low	Low	High
Shinh et al. (2017) [33]	Moderate	Moderate	Low	Moderate	Low	Moderate
Stockstill et al. (2011) [34]	Moderate	Moderate	Low	Moderate	Low	Moderate

## Discussion

Evaluations of orthodontic residency programs emphasize the importance of balanced approaches that integrate evidence-based education with hands-on clinical training. Effective curricula often blend traditional didactic learning with innovative, interactive teaching methods, ensuring that residents not only acquire theoretical knowledge but also develop practical skills essential for clinical competence [34]. Programs that successfully combine structured assessments, real-world clinical exposure, and up-to-date scientific instruction are better equipped to address the diverse learning needs of residents, helping them navigate complex patient cases with confidence [34].

### Structured clinical assessments

Structured clinical assessments, such as the Mini-Clinical Evaluation Exercise (Mini-CEX) and Objective Structured Clinical Examination (OSCE), play an essential role in orthodontic postgraduate education by ensuring residents develop key clinical competencies. These approaches provide structured feedback that improves diagnostic accuracy, patient communication, and procedural skills, reinforcing residents'ability to make evidence-based clinical decisions [9, 10, 15]. While Mini-CEX is used as a work-based assessment in real clinical settings, OSCEs offer a standardized evaluation environment, ensuring consistency across trainees. The effectiveness of both methods depends on faculty calibration, feedback quality, and logistical feasibility, with each having strengths and limitations in training and assessment [11, 22].

The Mini-CEX allows for direct observation of clinical encounters, enabling faculty to provide immediate and structured feedback to residents. Jamenis et al. (2020) found that Mini-CEX improved orthodontic residents'technical and decision-making skills, although effectiveness varied depending on assessment frequency and feedback depth [18, 28]. While students generally appreciate the formative nature of Mini-CEX, concerns remain about evaluation consistency and the stress associated with direct observation [8]. Faculty training plays a crucial role in ensuring that assessments remain objective and constructive, as inconsistencies in evaluation approaches can impact students'learning experiences [22].

The OSCE provides a standardized, structured evaluation of clinical competence, testing students across multiple stations designed to assess distinct skills. Fields et al. (2007) highlight its role in improving diagnostic accuracy, procedural proficiency, and communication skills through simulated patient interactions [15]. Unlike Mini-CEX, which evaluates students in live clinical environments, OSCEs ensure consistency across all trainees, mitigating variability caused by differences in patient cases or instructor expectations [9]. Additionally, structured assessments such as the OSCE have been linked to higher student confidence and motivation, as residents receive immediate, actionable feedback that informs their clinical practice [22]. However, the OSCE's effectiveness is contingent on examiner calibration and case standardization, as variability in scoring can still occur based on case complexity and faculty interpretations.

Despite their benefits, structured assessments like OSCEs present several challenges. Their design and implementation are resource-intensive, requiring faculty training, standardized patient recruitment, and significant administrative support, which can strain educational programs [15]. Furthermore, OSCEs'high-stakes nature can induce anxiety and stress among students, potentially impacting performance, particularly in timed or interactive components [22]. Additionally, while structured assessments provide quantifiable evaluation metrics, their reliance on predefined tasks may limit the assessment of complex clinical reasoning skills, which are better captured in real-time clinical settings [9].

Similarly, feedback-oriented learning approaches—whether within Mini-CEX or OSCE—are critical to skill development but introduce variability based on faculty expertise and feedback delivery. Studies indicate that residents who receive timely, personalized feedback perform better clinically, as they can identify areas for improvement and implement targeted learning strategies [22]. However, inconsistent feedback, overly critical assessments, or a lack of specificity can negatively impact confidence and engagement [22]. The effectiveness of feedback-driven assessments ultimately depends on faculty training, assessment standardization, and institutional support, ensuring that students receive actionable, meaningful guidance [9].

### Interactive learning approaches

Interactive learning methods, such as Scenario-Based Learning (SBL) and Problem-Based Learning (PBL), have gained prominence in orthodontic postgraduate education due to their ability to enhance critical thinking, clinical decision-making, and problem-solving skills [12, 23]. Both approaches emphasize active learning over passive knowledge acquisition, allowing students to develop their diagnostic and treatment planning abilities in a structured yet dynamic environment [27]. While SBL provides guided exposure to predefined clinical scenarios, PBL challenges students to independently navigate complex patient cases with minimal instructor intervention, promoting greater self-directed learning [20]. Studies have demonstrated that both methods contribute to improved preparedness for real-world clinical challenges, fostering confidence and adaptability among orthodontic residents [12, 27].

Both SBL and PBL have been shown to broaden clinical competence by immersing students in realistic patient care scenarios [11]. The structured nature of SBL allows residents to engage with interactive digital modules, integrating clinical videos, self-assessments, and evidence-based literature to guide decision-making [27]. This method is particularly effective for presenting complex orthodontic conditions that may not be frequently encountered in routine clinical practice, helping students develop diagnostic proficiency in controlled settings [25, 27]. PBL, by contrast, encourages self-directed exploration of patient cases, requiring students to formulate their own treatment plans and evaluate multiple possible solutions [12, 23]. This iterative learning process promotes deep engagement with theoretical knowledge while reinforcing its practical application in clinical scenarios.

The effectiveness of interactive learning approaches is further supported by their role in improving procedural confidence and decision-making skills. Orthodontic residents who engage in SBL-based clinical simulations report greater readiness for high-stakes patient management situations, particularly in orthodontic emergencies and multidisciplinary treatment planning [20]. Similarly, PBL fosters superior diagnostic reasoning and adaptability, as students are continuously challenged to assess their understanding and refine their treatment approaches based on evolving case information [23]. Additionally, PBL’s flexibility in non-traditional formats, such as distance learning and virtual case discussions, has demonstrated its capacity to maintain high levels of engagement and knowledge retention even in remote education settings [13].

Despite their benefits, both SBL and PBL present challenges in implementation. Technical difficulties, content quality concerns, and limited faculty-student interaction are commonly cited barriers in SBL, particularly when digital modules experience software glitches or inadequate instructional design [24, 27]. Likewise, PBL requires strong facilitator guidance, as unstructured learning environments can lead to student frustration and difficulty connecting different aspects of the material [12]. Additionally, PBL’s reliance on small-group participation and continuous reflection can create stress among students unfamiliar with self-directed learning, making adaptation to this method difficult for some learners [17, 23].

### Technology-enhanced learning

The use of technology-enhanced learning in orthodontic postgraduate education has expanded significantly, offering flexible, accessible, and interactive learning experiences that complement traditional clinical training [10]. Digital platforms, including Virtual Learning Environments (VLEs) and Distance Learning Seminars, have become essential in modern curricula, particularly in situations where direct clinical exposure is limited, such as during the COVID-19 pandemic or for geographically dispersed training programs [13, 32]. These approaches integrate self-directed learning, real-time instruction, and interactive digital tools, allowing residents to develop critical clinical competencies and theoretical knowledge in structured virtual settings [11].

Technology-driven learning environments provide structured content delivery through recorded lectures, case-based simulations, and interactive modules, reinforcing clinical concepts and procedural skills [7, 32]. Studies have shown that students engaging in digital learning platforms exhibit improved retention of orthodontic principles, particularly when multimedia elements such as video demonstrations and interactive assessments are incorporated [7]. These methods allow residents to revisit complex material at their own pace, enhancing long-term knowledge retention and procedural accuracy [32]. Furthermore, interactive virtual seminars facilitate real-time discussions, expert-led case reviews, and peer collaboration, fostering engagement and knowledge exchange in a remote setting [13].

Despite their advantages, technology-enhanced learning presents challenges, including technical barriers, reduced faculty-student interaction, and variability in student engagement [26, 33]. While asynchronous modules offer flexibility, students often report difficulty staying motivated without structured instructor guidance [32]. Similarly, live virtual sessions, while interactive, may be hindered by technological limitations, inconsistent internet access, and faculty adaptation challenges [33]. These obstacles highlight the need for careful integration of technology, ensuring that digital tools are used to enhance—not replace—hands-on clinical education.

### Educational strategies and student experience

The effectiveness of orthodontic postgraduate education is influenced by how students interact with different teaching methods, including their learning preferences, perceptions of instructional approaches, and experiences with assessment strategies [19]. Research indicates that orthodontic residents exhibit diverse learning styles, with a strong preference for visual and structured learning methods, emphasizing the importance of incorporating interactive, case-based, and multimodal teaching strategies [16, 21]. The way students engage with assessments and feedback mechanisms also plays a critical role in shaping their clinical competence and confidence in patient management [22, 29–31].

Studies suggest that visual and sequential learning styles are predominant among orthodontic residents, with students demonstrating higher engagement when presented with structured, step-by-step educational materials such as clinical videos, digital models, and case-based simulations [16]. The integration of interactive technologies and hands-on learning modules has been found to enhance student understanding and retention of complex orthodontic procedures, particularly when combined with structured assessment and feedback-oriented learning approaches [21]. Assessment strategies such as Mini-CEX and OSCEs have been shown to improve diagnostic reasoning and clinical decision-making skills, as they provide structured, immediate feedback that reinforces learning objectives [9, 15].

The role of feedback in student experience and performance is particularly significant, as residents who receive consistent, personalized feedback tend to perform better clinically and exhibit greater confidence in their decision-making abilities [22]. However, challenges arise when feedback is inconsistent or lacks specificity, leading to uncertainty in clinical skill development [22]. Additionally, structured assessments such as OSCEs, while effective in measuring clinical proficiency, can induce performance anxiety, particularly in high-stakes evaluation settings [15].

Despite these challenges, aligning educational strategies with student learning preferences enhances engagement, motivation, and clinical competency development. Programs that integrate case-based learning, structured assessment tools, and tailored feedback mechanisms provide students with a comprehensive and adaptable learning experience, reinforcing both technical skills and critical thinking in orthodontic practice.

### Integrating evidence-based learning into orthodontic practice

Assessment methods and structured educational interventions play a critical role in developing clinical competence among orthodontic postgraduate students. Various instructional approaches, including structured feedback, standardized assessments, and interactive learning methods, have been shown to improve students'ability to apply theoretical knowledge to clinical practice [15]. However, significant variability exists across orthodontic programs in terms of assessment formats, grading criteria, and feedback mechanisms, which creates inconsistencies in how clinical skills are evaluated [9]. Addressing these inconsistencies through more standardized, competency-based assessment frameworks may improve the reliability of postgraduate training and ensure that students meet defined educational benchmarks before independent practice [22].

Structured assessments have been widely adopted in orthodontic education due to their ability to provide timely, targeted feedback that enhances students'diagnostic reasoning and procedural skills [22]. Studies indicate that frequent, structured feedback through these methods helps students identify weaknesses and refine their clinical decision-making abilities, making them more confident in their ability to manage complex patient cases [15, 22]. However, inconsistencies in faculty expectations and grading rubrics limit the generalizability of these assessments across different training institutions [9]. Implementing faculty development programs to standardize evaluation methods could improve inter-rater reliability and ensure that students receive consistent, actionable feedback that aligns with clinical practice expectations [15].

Beyond assessment strategies, interactive and digital learning interventions have been increasingly integrated into orthodontic education. Simulation-based learning and case-based discussions allow students to engage with complex patient scenarios in a controlled environment, enhancing their critical thinking and adaptability [9]. Additionally, digital learning tools, including virtual reality simulations and self-assessment modules, have been reported to increase engagement and improve knowledge retention, particularly when used in combination with traditional instructional methods [22]. While these tools have demonstrated clear benefits, their effectiveness is heavily dependent on proper instructional design and faculty guidance, ensuring that they supplement rather than replace hands-on clinical experience [15].

A major challenge in orthodontic postgraduate education is the disparity in educational resources and assessment implementation across programs. Institutions with greater technological and faculty support tend to implement more sophisticated assessment and feedback models, whereas those with limited resources may rely on traditional, less interactive methods [9, 14]. To bridge this gap, promoting international collaboration and knowledge-sharing initiatives could facilitate the adoption of best practices across programs with varying levels of resources [6]. Developing global competency frameworks for orthodontic postgraduate education would also provide a structured approach to assessment, ensuring a more equitable and rigorous evaluation of clinical training outcomes [15].

While structured assessments and digital learning interventions contribute to improved orthodontic training, ongoing curriculum evaluation remains necessary to ensure that these methods remain aligned with evolving clinical and educational needs. Future research should explore the long-term impact of various assessment models on clinical competence and patient outcomes, helping to refine and optimize orthodontic education [9]. Continuous improvement in evaluation strategies, faculty training, and resource allocation will help ensure that postgraduate orthodontic programs equip residents with the necessary skills to provide high-quality patient care [15].

### Recommendations and future research

To enhance the consistency and effectiveness of orthodontic postgraduate education, programs should adopt standardized, competency-based frameworks that align instructional content and assessment strategies with clearly defined clinical outcomes. Studies included in this review highlighted that structured assessments such as OSCEs and Mini-CEX are widely used to evaluate procedural skills, diagnostic reasoning, and communication competencies [9, 15, 22]. However, inconsistent grading criteria and uncalibrated feedback practices across institutions undermine the reliability of these tools [9, 22]. To address this, institutions should invest in faculty development programs that focus on assessment calibration, feedback delivery, and the use of validated evaluation instruments. Evidence suggests that regular faculty training can improve inter-rater reliability and ensure a more consistent and supportive learning environment [14, 22].

In addition, curriculum design should integrate interactive learning approaches—such as PBL, SBL, and digital simulation modules—that promote self-directed learning, critical thinking, and diagnostic accuracy [12, 20, 23, 27]. These strategies have been shown to improve student engagement and clinical preparedness, especially when supported by structured feedback and realistic case exposure [20, 27]. Given the increasing role of technology in orthodontic training, virtual learning environments and distance education modules have been reported to enhance knowledge retention and flexibility [13, 32]. However, their effectiveness depends on instructional design and faculty facilitation, as unstructured or poorly supported implementations may limit student motivation and interaction [7, 32]. These digital tools should complement, not replace, hands-on clinical training.

Efforts to standardize curricula must also account for disparities in institutional resources and infrastructure. Institutions with greater technological capacity and faculty support tend to offer more interactive and feedback-driven programs, while resource-limited settings often rely on more traditional instructional approaches [9, 14]. Educational policymakers and professional associations should consider developing global competency benchmarks and adaptable assessment guidelines to ensure minimum standards while allowing contextual flexibility [15]. Initiatives promoting international collaboration—such as faculty exchanges, shared repositories of learning materials, and cross-institutional assessment tools—could help bridge gaps between programs and enhance educational equity [6].

Future research should address critical evidence gaps identified in this review. Longitudinal studies are needed to evaluate the impact of specific instructional strategies on clinical competence, professional development, and patient care quality [9, 22]. Multi-institutional research involving diverse geographic and institutional contexts would strengthen generalizability and clarify which educational interventions are most effective across settings [22, 29]. Additionally, establishing consensus definitions and validated metrics for clinical competence is essential for enabling consistent measurement and informing future curriculum reforms [9, 22, 29].

### Strengths and limitations

This study offers a comprehensive evaluation of diverse educational methods, highlighting the adaptability of postgraduate orthodontic programs in optimizing clinical competence. By including studies from multiple geographic regions, this review enhances external validity, providing insights into how differences in educational standards influence clinical training and patient outcomes. The use of robust assessment tools, including the NOS for study quality and the GRADE approach for evaluating evidence strength, further reinforces the reliability of findings. High NOS scores in selection and outcome domains indicate that most included studies had well-defined sample groups and reliable outcome measures, contributing to the overall strength of this review.

However, several limitations must be acknowledged. The variability in study design and methodological quality reduces the ability to draw definitive conclusions about the impact of specific educational interventions on clinical competence. Lower comparability scores on the NOS indicate gaps in controlling for confounding factors, which affects internal validity. Furthermore, the GRADE assessments frequently classified evidence quality as moderate, reflecting concerns related to bias, inconsistency, and imprecision, underscoring the need for more rigorous study designs with standardized outcome measurements. The interpretation of findings was further influenced by the risk of bias across studies, as assessed through the NOS and GRADE frameworks. While most studies demonstrated strong selection criteria, inconsistencies in study design and assessment tools suggest that some conclusions should be interpreted with caution, particularly where outcome measures lacked precision.

Another limitation lies in the inconsistent definition and measurement of clinical competence across the included studies. While some used structured assessments such as OSCEs and Mini-CEX to evaluate procedural skill, communication, and diagnostic accuracy, others relied on self-report measures or academic performance. A few studies included broader definitions involving clinical judgment or integration of knowledge into practice. This lack of standardization complicates the synthesis of findings and limits the ability to draw direct comparisons across programs. Establishing shared definitions and validated measurement tools is essential for improving the comparability and generalizability of future research in this area.

The findings of this review must be interpreted in the context of substantial heterogeneity among the included studies. Differences in study design, assessment formats, institutional resources, and educational frameworks limited the ability to make direct comparisons or to conduct meta-analytic synthesis. While this diversity reflects the real-world complexity of postgraduate orthodontic education, it also constrains the generalizability of specific instructional or assessment strategies. For example, a given teaching intervention may yield positive outcomes in one setting but may not be replicable in another due to differences in faculty expertise, student demographics, or institutional infrastructure. Moreover, inconsistencies in reporting outcome measures and feedback protocols further complicate the ability to draw unified conclusions about educational effectiveness. Acknowledging this variability is essential for understanding the nuanced and context-dependent nature of clinical education, and for encouraging adaptable, rather than prescriptive, curriculum development.

## Conclusion

This systematic review examined how clinical knowledge is delivered in orthodontic postgraduate programs and how instructional strategies relate to the development of clinical competence. Across diverse contexts, structured assessment methods, interactive learning approaches, and technology-enhanced instruction were frequently associated with improved clinical performance and decision-making. However, variability in implementation, assessment formats, and faculty training contributed to inconsistent outcomes. These findings suggested that postgraduate orthodontic programs may benefit from adopting adaptable, evidence-based teaching models that support clinical skill development while accommodating institutional differences in resources and structure.

## Data Availability

No datasets were generated or analysed during the current study.

## References

[1] Allanqawi T, Alkadhimi A, Fleming PS. Postgraduate orthodontic education: an international perspective on content and satisfaction levels. J World Fed Orthod. 2023;12(6):239–44. 37739847 10.1016/j.ejwf.2023.08.004

[2] Ono T, Pangrazio-Kulbersh V, Perillo L, Artese F, Czochrowska E, Darendeliler MA, et al. World Federation of Orthodontists guidelines for postgraduate orthodontic education. J World Fed Orthod. 2023;12(2):41–9. 36964071 10.1016/j.ejwf.2023.03.002

[3] Alam MK, Abutayyem H, Kanwal B, Shayeb MAL. Future of orthodontics—a systematic review and meta-analysis on the emerging trends in this field. J Clin Med. 2023;12(2):532. 36675459 10.3390/jcm12020532 PMC9861462

[4] Wells GA, O’Connell D, Peterson J, Welch V, Losos M, Tugwell P. The Newcastle-Ottawa Scale (NOS) for assessing the quality of nonrandomised studies in meta-analyses. 2021. Available from: https://www.ohri.ca/programs/clinical_epidemiology/oxford.asp. Cited 2025 Feb 9.

[5] Cochrane Training. GRADE approach. 2024. Available from: https://training.cochrane.org/grade-approach. Cited 2024 Jul 16.

[6] Cure R. Interprofessional education in an orthodontic outreach training centre. Prim Dent J. 2016;5(4):63–9. 28107137 10.1308/205016816820209451

[7] Aly M, Willems G, Van Den Noortgate W, Elen J. Effect of multimedia information sequencing on educational outcome in orthodontic training. Eur J Orthod. 2012;34(4):458–65. 21734254 10.1093/ejo/cjr036

[8] Al-Jewair T, Kumar S. Review and application of the Mini-Clinical Evaluation Exercise (Mini-CEX) in advanced orthodontic education: a pilot study. J Dent Educ. 2019;83(11):1332–8. 31332042 10.21815/JDE.019.131

[9] Allareddy V, Rengasamy Venugopalan S, Nalliah RP, Caplin JL, Lee MK, Allareddy V. Orthodontics in the era of big data analytics. Orthod Craniofac Res. 2019;22(1):8–13. 10.1111/ocr.12279. PMID: 31074158. 10.1111/ocr.12279 31074158

[10] Aly M, Willems G, Carels C, Elen J. Instructional multimedia programs for self-directed learning in undergraduate and postgraduate training in orthodontics. Eur J Dent Educ. 2003;7(1):20–6. 12542685 10.1034/j.1600-0579.2003.00263.x

[11] Arunachalam S, GVD H, Sharan J, Ghandikota A, Ram RR, Praveen G. Orthodontic postgraduate students’ perception on didactics and clinical training during the COVID-19 crisis. J Indian Orthod Soc. 2021;55(3):243–50.

[12] Bearn DR, Chadwick SM. Problem-based learning in postgraduate dental education: a qualitative evaluation of students’ experience of an orthodontic problem-based postgraduate programme. Eur J Dent Educ. 2010;14(1):26–34. 20070796 10.1111/j.1600-0579.2009.00588.x

[13] Bednar ED, Hannum WM, Firestone A, Silveira AM, Cox TD, Proffit WR. Application of distance learning to interactive seminar instruction in orthodontic residency programs. Am J Orthod Dentofacial Orthop. 2007;132(5):586–94. 18005831 10.1016/j.ajodo.2007.06.008

[14] Burk T, Orellana M. Assessment of graduate orthodontic programs in North America. J Dent Educ. 2013;77(4):463–75. 23576592

[15] Fields HW, Rowland ML, Vig KWL, Huja SS. Objective structured clinical examination use in advanced orthodontic dental education. Am J Orthod Dentofacial Orthop. 2007;131(5):656–63. 17482087 10.1016/j.ajodo.2007.01.013

[16] Hughes JM, Fallis DW, Peel JL, Murchison DF. Learning styles of orthodontic residents. J Dent Educ. 2009;73(3):319–27. 19289721

[17] Ireland AJ, Atack NE, Sandy JR. Experiences of Wiki topic teaching in postgraduate orthodontics: what do the learners think? Eur J Dent Educ. 2013;17(1):e109–13. 23279397 10.1111/j.1600-0579.2012.00769.x

[18] Jamenis SC, Pharande S, Potnis S, Kapoor P. Use of mini clinical evaluation exercise as a tool to assess the orthodontic postgraduate students. J Indian Orthod Soc. 2020;54(1):39–43.

[19] Johnson King O, Ryan F, Cunningham S. Postgraduate student perceptions of face-to-face and distance education in orthodontics: a cross-sectional qualitative study. J Orthod. 2022;49(3):280–7. 35302421 10.1177/14653125221077108 PMC9421199

[20] Kashif M. Postgraduate educational programs preferences in Pakistan and factors affecting it: a cross-sectional survey conducted among dental students of Karachi, Pakistan. Pak J Med Res. 2020 May 2.

[21] Lishoy R, Bhushan J, Vanessa V, Hidhaya SK, Rashi L. An assessment of common concerns of 2nd year postgraduate students pursuing M.D.S in orthodontics and dentofacial orthopedics due to the COVID-19 lockdown. In: IP Indian J Orthod Dentofacial Res. 2020. p. 184–90. Available from: https://ijodr.com/article-details/12085. Cited 2024 Sep 19.

[22] Madhan B, Rajpurohit AS, Gayathri H. Attitudes of postgraduate orthodontic students in India towards patient-centered care. J Dent Educ. 2011;75(1):107–14. 21205735

[23] Masella RS, Meister M. Challenges to postdoctoral orthodontic education. Am J Orthod Dentofacial Orthop. 2002;122(2):221–5. 12165779 10.1067/mod.2002.125564

[24] Mcdonald JP, Adamidis JP, Eaton KA, Seeholzer H, Sieminska-Piekarczyk B. A survey of postgraduate (specialist) orthodontic education in 23 European countries. J Orthod. 2000;27(1):83–98. 10790453 10.1093/ortho/27.1.92

[25] Mohan A, Babu H. A survey on use of digital study models in postgraduate orthodontic programs. Int J Pharm Res. 2020;12(4):2101–7.

[26] Nakornnoi T, Chantakao C, Luangaram N, Janbamrung T, Thitasomakul T, Sipiyaruk K. Perceptions of orthodontic residents toward the implementation of dental technologies in postgraduate curriculum. BMC Oral Health. 2023;23(1):625. 37658317 10.1186/s12903-023-03327-x PMC10474673

[27] Naser-ud-Din S. Introducing scenario-based learning interactive to postgraduates in UQ orthodontic program. Eur J Dent Educ. 2015;19(3):169–76. 25212808 10.1111/eje.12118

[28] Oliver GR, Lynch CD, Fleming PS. What I wish I’d learned as an orthodontic trainee: an online survey of British Orthodontic Society members concerning postgraduate training experiences. J Orthod. 2020;47(2):116–28. 32052682 10.1177/1465312520904367 PMC7498909

[29] Papageorgiou SN, Koletsi D, Patcas R, Will LA, Eliades T. Knowledge of postgraduate dental students on evidence-based dentistry and research methodology: an international survey. Oral Health Prev Dent. 2020;18(1):873–9. 33215479 10.3290/j.ohpd.a45404 PMC11654606

[30] Papageorgiou SN, Koletsi D, Patcas R, Seehra J, Cobourne MT, Will LA, et al. Knowledge of evidence-based research methodology amongst orthodontic postgraduate residents in four universities: an international survey. Int Orthod. 2022;20(1):100609. 35093271 10.1016/j.ortho.2022.100609

[31] Polychronopoulou A, Eliades T, Taoufik K, Papadopoulos MA, Athanasiou AE. Knowledge of European orthodontic postgraduate students on biostatistics. Eur J Orthod. 2011;33(4):434–40. 21076157 10.1093/ejo/cjq098

[32] Shah R, Cunningham SJ. Implementation of the virtual learning environment into a UK orthodontic training programme: the postgraduate and lecturer perspective. Eur J Dent Educ. 2009;13(4):223–32. 19824959 10.1111/j.1600-0579.2009.00579.x

[33] Shinh AS, Guram G, Shaik JA, Singla P, Kaur A, Maheshwari K. Challenges encountered during postgraduate program in orthodontics: an online survey. J Indian Orthod Soc. 2017;51(4):258–63.

[34] Stockstill J, Greene CS, Kandasamy S, Campbell D, Rinchuse D. Survey of orthodontic residency programs: Teaching about occlusion, temporomandibular joints, and temporomandibular disorders in postgraduate curricula. Am J Orthod Dentofacial Orthop. 2011;139(1):17–23. 21195272 10.1016/j.ajodo.2010.10.001

